# Detection of circulating tumor DNA in plasma of patients with primary CNS lymphoma by digital droplet PCR

**DOI:** 10.1186/s12885-024-12191-z

**Published:** 2024-04-02

**Authors:** Yujie Zhong, Geok Wee Tan, Johanna Bult, Nick Veltmaat, Wouter Plattel, Joost Kluiver, Roelien Enting, Arjan Diepstra, Anke van den Berg, Marcel Nijland

**Affiliations:** 1 Department of Hematology, University of Groningen, University Medical Center Groningen, Groningen, The Netherlands; 2 Department of Pathology and Medical Biology, University of Groningen, University Medical Center Groningen, Groningen, The Netherlands; 3 Department of Neurology, University of Groningen, University Medical Center Groningen, Groningen, The Netherlands; 4 Molecular Pathology Unit, Cancer Research Centre, Institute for Medical Research, Kuala Lumpur, Malaysia

**Keywords:** Circulating tumor DNA, Primary central nervous system lymphoma, MYD88, CD79B, Liquid biopsy

## Abstract

**Background:**

Primary central nervous system lymphoma (PCNSL) are rare mature B-cell lymphoproliferative diseases characterized by a high incidence of MYD88 L265P and CD79B Y196 hotspot mutations. Diagnosis of PCNSL can be challenging. The aim of the study was to analyze the detection rate of the MYD88 L265P and CD79B Y196 mutation in cell free DNA (cfDNA) in plasma of patients with PCNSL.

**Methods:**

We analyzed by digital droplet PCR (ddPCR) to determine presence of the MYD88 L265P and CD79B Y196 hotspot mutations in cfDNA isolated from plasma of 24 PCNSL patients with active disease. Corresponding tumor samples were available for 14 cases. Based on the false positive rate observed in 8 healthy control samples, a stringent cut-off for the MYD88 L265P and CD79B Y196 mutation were set at 0.3% and 0.5%, respectively.

**Results:**

MYD88 L265P and CD79B Y196 mutations were detected in 9/14 (64%) and 2/13 (15%) tumor biopsies, respectively. In cfDNA samples, the MYD88 L265P mutation was detected in 3/24 (12.5%), while the CD79B Y196 mutation was not detected in any of the 23 tested cfDNA samples. Overall, MYD88 L265P and/or CD79B Y196 were detected in cfDNA in 3/24 cases (12.5%). The detection rate of the combined analysis did not improve the single detection rate for either MYD88 L265P or CD79B Y196.

**Conclusion:**

The low detection rate of MYD88 L265P and CD79B Y196 mutations in cfDNA in the plasma of PCNSL patients argues against its use in routine diagnostics. However, detection of MYD88 L265P by ddPCR in cfDNA in the plasma could be considered in challenging cases.

**Supplementary Information:**

The online version contains supplementary material available at 10.1186/s12885-024-12191-z.

## Background

Primary central nervous system lymphoma (PCNSL) is a mature lymphoproliferative disease that arises in the central nervous system [1]. PCNSL is a rare condition in immunocompetent patients, accounting for 4% of all intracranial neoplasms and 4–6% of all extranodal mature B-cell lymphoproliferative diseases (LPD). Nevertheless, the incidence of PCNSL has increased in recent years, particularly among individuals over 60 years, with an incidence rate of 0.5 per 100,000 per year [2]. PCNSL is characterized by recurrent genomic features, including loss of HLA loci located at 6p21.32–p25.3, amplification of 9p24 including the PD-L1 locus, and MYD88 L265P and CD79B Y196 hotspot mutations [3]. The MYD88 L265P and CD79B Y196 hotspot mutations are present in 60% and 64% of PCNSL patients, respectively [3–5]. CD79B Y196 mutations co-occurred in about 35% of MYD88 L265P positive PCNSL cases [3, 6]. MYD88 L265P and CD79B Y196 mutations have not been described in any malignancy other than B-cell lymphomas and are observed in less than 0.01% of the general population [7–9].

Diagnosis of PCNSL can be challenging. Cytomorphology and flow cytometry analysis of cerebral spinal fluid (CSF) is sufficient for a reliable diagnosis only in about 15% of cases [10]. In the remaining patients, a tumor biopsy is necessary, which carries the risk of complications and is not always feasible due to the anatomical localization of the tumor, poor performance status of the patient, or not being eligible for surgery because of multiple comorbidities [11]. Response assessment relies on imaging studies. However, MRI scans often cannot differentiate between active tumor and scar tissue, and 18 F-FDG/PET-scans are ineffective in assessing CNS lymphomas [11]. Hence, there is a need for non-invasive tools to aid in diagnosis and help in response assessment.

Cell free DNA (cfDNA) refers to all non-encapsulated DNA that enters the circulating system. In patients with cancer, a proportion of the cfDNA originates from tumor cells and is referred to as circulating tumor DNA (ctDNA). Several studies have demonstrated the value of ctDNA in diagnosis, prognosis, and monitoring of disease activity in systemic diffuse large B-cell lymphoma (DLBCL) [12].

Recently, digital droplet PCR (ddPCR) analysis of cfDNA isolated from CSF [13] and targeted next generation sequencing of plasma derived cfDNA [14] was presented as a promising biomarker for PCNSL. Digital droplet PCR (ddPCR) is a robust method, for the detection of mutations even at low variant allele frequencies and does not require a bio-informatics pipeline but can be analyzed instantaneously. Analysis of ctDNA from CSF of PCNSL patients had a high specificity and was correlated with outcome [13]. In PCNSL patients whose brain lesions are surgically inaccessible, detection of hotspot mutations in plasma-derived cfDNA might aid in diagnosis [13–15].

In this study, we aimed to investigate the clinical value of detecting the combination of MYD88 L265P and CD79B Y196 hotspot mutations in cfDNA isolated from plasma of patients with active disease by ddPCR.

## Materials and methods

### Patients

We enrolled a total of 24 PCNSL patients based on availability of plasma samples in the OncoLifes biobank and active measurable disease at the time of sample collection [16]. Histology diagnosis was confirmed in 23 out of 24 patients by an experienced hematopathologist (AD) based on available immunohistochemically stained tissue sections. For 14 out of these 23 cases, we were able to retrieve formalin-fixed paraffin-embedded (FFPE) tissue specimens acquired at the time of diagnosis, enabling subsequent ddPCR analysis. In the remaining case, the diagnosis PCNSL was confirmed by the combination of cytomorphology and flow cytometry analysis of the spinal fluid. All patients had active disease indicated by contrast enhancing lesions at the MRI scan at the time of plasma collection. White blood cells (WBC) of eight healthy subjects were used for DNA isolation and for determining cutoff values for ddPCR. The study was approved by the Medical Ethics Review Board of the University Medical Center Groningen. Informed consent was obtained from all included patients.

### Sample collection and DNA extraction

Plasma samples were retrieved retrospectively from the Oncolifes biobank. In brief, blood samples were collected either in EDTA (*n* = 7) or Streck tubes (*n* = 17). Plasma was isolated using standard laboratory procedures by centrifugation at 4℃, 2000 g for 10 min within eight hours of collection. Plasma was aliquoted in batches of 1 ml and stored at -80℃ until further use. CfDNA was extracted from 2 to 4 ml plasma using the QIAamp circulating Nucleic Acid Kit following the protocol provided by the manufacturer (Qiagen, Hilden, Germany). QIAamp DNA FFPE Tissue Kit Genomic DNA (gDNA) was used to extract DNA from two to four 5 μm FFPE tissue sections according to the protocol of the manufacturer (Qiagen, Hilden, Germany). DNA of healthy controls was isolated using ReliaPrep Large Volume HT gDNA Isolation kit (Promega, Madison, WI, USA) using the Hamilton StarPlus robotic system (Hamilton, Reno, NV, USA). DNA concentrations were determined using the Qubit dsDNA HS Assay Kit (Thermo Fisher Scientific, Carlsbad, CA, USA) according to the protocol provided by the manufacturer.

### Digital droplet PCR

The ddPCR was performed following the protocol provided by the manufacturer using assays specific for the hotspots of MYD88 L265P and CD79B Y196 (Bio-Rad Laboratories, Hercules, USA). The ddPCR assay for MYD88 targets the c.794T > C p.L265P variant and the drop-off assay for CD79B Y196 targets c.586T > C p.Y196H, c.586T > G p.Y196D, c.587 A > C p.Y196S, c.587 A > G p.Y196C, and c.587 A > T p.Y196F variants. Droplets were generated with the QX200 droplet generator. The amplification reaction was initiated by an enzyme activation step for 10 min, which was followed by 39 cycles of 94 °C for 30 s, 55 °C (MYD88 L265P) or 53 °C (CD79B Y196) for 1 min and a final incubation at 98 °C for 10 min. Droplets were loaded into the QX200 droplet reader and analyzed by QuantaSoft version 1.0 (Bio-Rad Laboratories). For each experiment we included a water control to monitor environmental contamination. DNA of SUDHL10 and OCI-ly3 were used as wild type controls for MYD88 L265P and CD79B Y196 respectively. DNA isolated from OCI-ly3 was used as a positive control for the MYD88 L265P assay and DNA of a patient sample tested positive in the routine diagnostic test was used as a positive control for the CD79B Y196 assay. For the MYD88 L265P test, the ddPCR reaction wells clustered into four groups based on the fluorescence signal, wild type (HEX positive), mutant (FAM positive), double positive (both HEX and FAM), and no-template (double negative). For CD79B Y196, the wells clustered into three groups, wild type (double positive), mutant (FAM positive), and no-template (double negative). The variant allele frequency (VAF) was calculated by dividing the total number of FAM positive mutant droplets over the total number of filled droplets. To determine the linear range of the ddPCR assays we made a serial dilution of mutant DNA (OCI-ly3 for MYD88 L265P and the patient sample for CD79B Y196) in a background of wild type DNA (SUDHL10 for MYD88 L265P and OCI-ly3 for CD79B Y196). We aimed to mix the samples to achieve VAFs of 10%, 5%, 1%, 0.5%, 0.3%, 0.1%. Three independent dilution series were made for both assays and analyzed by ddPCR.

### Cutoff

To determine the rate of false positive droplets of the two ddPCR assays, we amplified genomic DNA isolated from WBC of eight healthy control subjects. Each sample was analyzed at three different concentrations, resulting in ddPCR results for 24 independent wells. These data were used to set a cutoff value for calling a patient sample positive. For the patient samples we aimed to analyze a minimum of 500 filled droplets in 2 to 5 independent wells. In addition, we applied a cutoff of at least 6 mutant positive droplets. Cases with a total number of filled droplets < 500 were defined as being inconclusive, due to low sensitivity.

### Statistical analysis

Statistical analyses applied were descriptive in nature. Differences in cfDNA yields EDTA- and Streck- tubes were tested using the nonparametric Mann-Whitney test. Analyses were done using GraphPad Prism Software v 8.0. and IBM SPSS Statistics 23. P-values < 0.05 were considered significant.

## Results

### Patient characteristics

The median age of patients was 66 years (range 41–75 years) with a slight female preponderance (13 females). Plasma samples were obtained during active disease, at diagnosis (*n* = 14), partial remission (*n* = 8) or progressive disease (*n* = 2).

### Total amount of cell free DNA

The median cfDNA yields was 14 ng per ml (range 4.4 to 65) of plasma. There was no significant difference between the cfDNA yields in plasma obtained from EDTA- and Streck-tubes (data not shown, *p* = 0.56) (Figure S1).

### ddPCR results

**Fig. 1:**
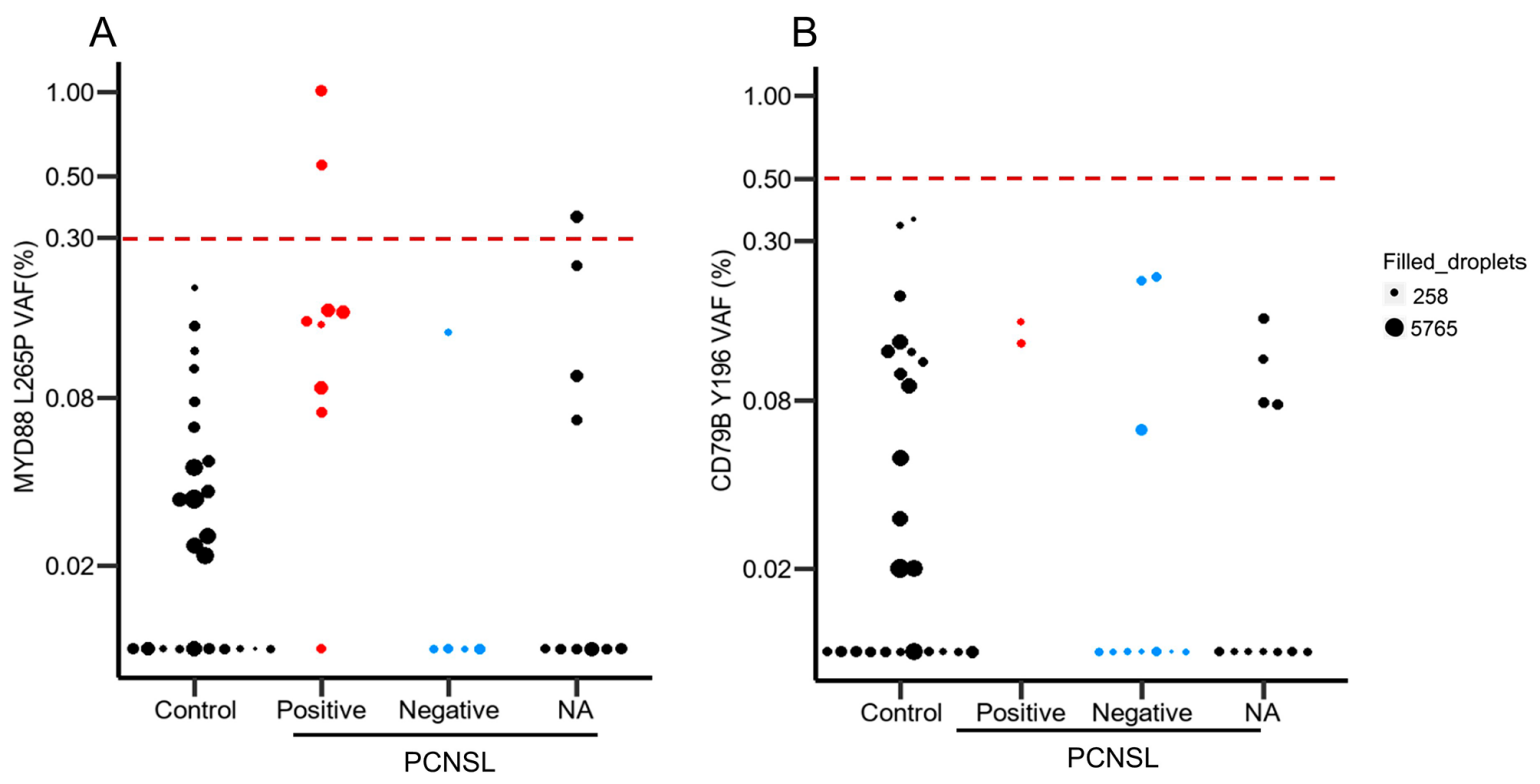
Variant alle frequency (VAF) of MYD88 L265P and CD79B Y196 variants in. (A) In patients the VAF of MYD88 L265P ranged from 0–1% and in controls it ranged from 0–0.20%. Cut-off value was set as 0.3%. (B) In patients the VAF of CD79B Y196 ranged from 0–0.22% and in controls it ranged from 0–0.36%. Cut-off value was set as 0.5%. X-axis shows types of FFPE tissue, being controls or PCNSL. PCNSL cases are divided based on the MYD88 or CD79B ddPCR results of the tissue samples as positive, negative, or data not available (NA). Dot sizes indicate the actual total filled droplets (mutation+/wildtype+) of each cfDNA sample, ranging from 258 to 5765 filled droplets. There are 5 samples with a total number of filled droplets < 500 which were defined as inconclusive (Table S3)

Efficiency and linear range of both ddPCR assays was tested using serial dilutions by mixing positive and negative DNA samples (Table S1 and Table S2). For MYD88 L265P the dilution series showed a clear linear pattern ranging from 14 to 0.25%. For CD79B Y196 the dilution series showed a linear range from 10 to 0.5% (Figure S2). To determine the cutoff values for both ddPCR assays we analyzed WBC control samples (Fig. 1). Based on the VAF range observed, we determined the cutoff value for MYD88 L265P at 0.3% and for CD79B Y196 at 0.5% (Fig. 1).

**Fig. 2:**
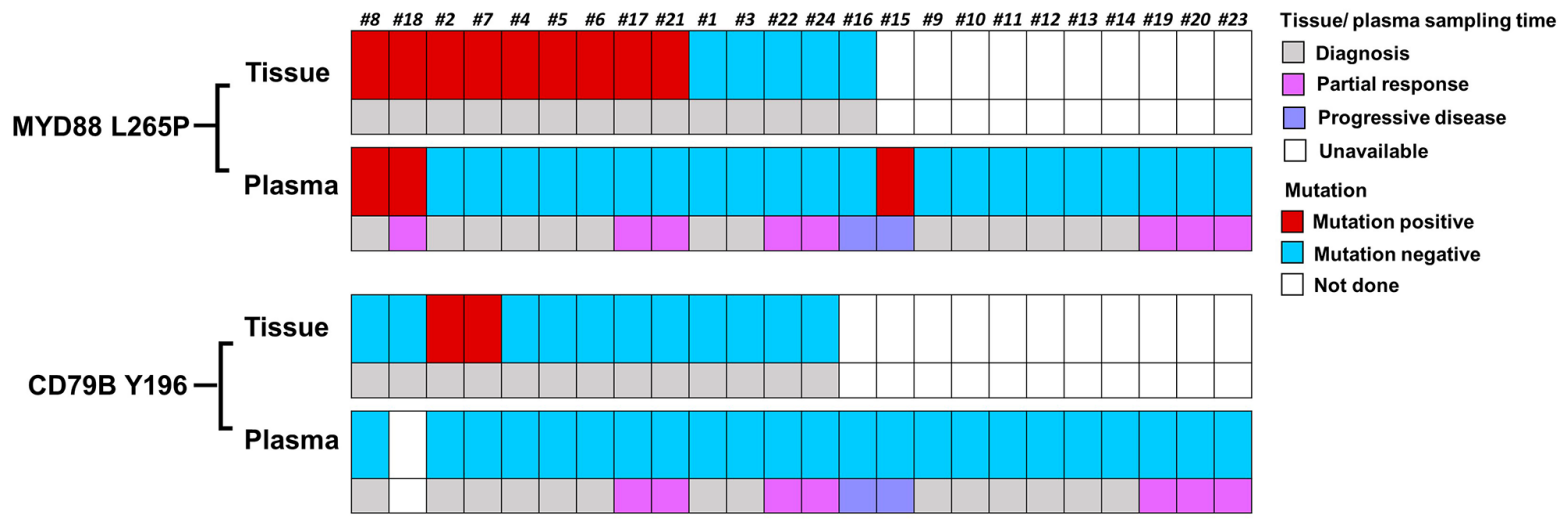
Digital droplet PCR results of MYD88 L265P and CD79B Y196 in patients with primary CNS lymphoma

MYD88 L265 were detected in 9/14 (64%) tissue samples and in 3/24 (12.5%) cfDNA samples. VAF of cfDNA ranged from 0 to 1% (median 0.065%) (Table S3). Two out of three positive cfDNA samples were also positive in tissue, while no tissue was available for the 6th patient (#15). The percentage of positive plasma samples in PCNSL patients is 12.5%. No obvious correlations were observed with disease status (Fig. 2).

For samples with sufficient cfDNA we next tested the presence of CD79B Y196 mutations (Fig. 2). This revealed mutations in 2/13 (15%) tissue samples and in 0/23 plasma cfDNA samples. No CD79B Y196 were observed in PCNSL plasma samples, also not in the two patients with a confirmed CD79B Y196 in the corresponding tissue sample. An overview of the ddPCR results per patient is shown in Table S3.

## Discussion

In this study, we investigated the feasibility of detecting MYD88 L265P and CD79B Y196 as a biomarker in cfDNA in plasma samples of PCNSL patients. While the MYD88 L265P were detected at high frequencies in tissue samples, this was limited in plasma samples (12.5%). CD79B Y196 and MYD88 L265P mutations have been described to co-occur in about 20% of PCNSL [3, 6]. In our study, the CD79B Y196 mutation co-occurred with the MYD88 L265P mutation in 2 out of 13 tissue samples analyzed for both variants, but none of them was detected in plasma. So, the added value of detecting the CD79B Y196 mutation was limited in our patient cohort.

The frequency of MYD88 L265P in cfDNA in the plasma as observed in our study is relatively low. In previous PCNSL studies, MYD88 L265P mutations were detected in 4% (1/23), 35% (6/17) and 57% (8/14) of the plasma samples [14, 17, 18]. These differences in positive rate might have been caused by differences in cut-off values applied to call a sample positive in combination with the number of unique DNA copies analyzed. In these studies, the MYD88 L265P VAF ranged from 0.01 to 2.3% (mean VAF 0.2%∼0.3%) [14, 17, 18]. We used a more stringent cut-off of 0.3% based on the false positive rate observed in healthy control samples, to avoid false positive results. A stringent cut-off value is critical for clinical decisions, especially in cases for which the prior knowledge of the MYD88 L265P status in the tumor is unknown.

In comparison to DLBCL, the overall positive rate of detecting somatic mutations in cfDNA in plasma of PCNSL patients is lower [14, 17, 19]. This difference might be caused by the difference in metabolic tumor volume as measured by 18 F-FDG PET scans. In DLBCL the ctDNA level in plasma was shown to be related to the total metabolic tumor volume [19]. However, PCNSL patients usually have relatively low tumor volumes [20]. In addition, no associations have been reported between plasma ctDNA levels and tumor burden in PCNSL. This difference might be related to the blood-brain barrier which restricts the exit of cells and soluble factors, including ctDNA, from the CNS into the blood.

In contrast to our plasma analysis, studies have shown that the VAF of MYD88 L265P in the CSF of PCNSL ranges from 2.6% to more than 90%, and that positive results can be obtained in about 50% of PCNSL patients [21–23]. This shows that the analysis of CSF strongly outperforms the plasma results. In this study, we did not have CSF of the PCNSL patients precluding a direct comparison between plasma and CSF results.

## Conclusions

In this study, the percentage of PCNSL cases with a MYD88 L265P mutation in cfDNA in the plasma as determined by ddPCR using a stringent cut off was 12.5%. Addition of CD79B Y196 did not improve detection rate. The low detection rate of MYD88 L265P and CD79B Y196 mutations in cfDNA in the plasma of PCNSL patients argues against the routine use. However, analysis of the MYD88 L265P in cfDNA in the plasma could be considered in challenging cases.

### Electronic supplementary material

Below is the link to the electronic supplementary material.

Supplementary Material 1: Table S1. ddPCR results of series dilution of MYD88 L265P; Table S2. ddPCR results of series dilution of CD79B Y196; Table S3. ddPCR results of healthy controls and patient samples; Figure S1. cfDNA yields of plasma samples from PCNSL patients; Figure S2. Series dilution VAF of MYD88 L265P and CD79B Y196.

## Data Availability

The authors confirm that the data supporting the findings of this study are available within the article and its supplementary data.

## Abbreviations

PCNSL: Primary central nervous system lymphoma
cfDNA: Cell free DNA
ctDNA: Circulating tumor DNA
ddPCR: Digital droplet PCR
CSF: Central spinal fluid
