# Supplementary material for: Detection of circulating tumor DNA in plasma of patients with primary CNS lymphoma by digital droplet PCR
Source: BMC Cancer. 2024 Apr 2;24:407. doi: 10.1186/s12885-024-12191-z (PMC10985975; doi:10.1186/s12885-024-12191-z)
Supplement: Supplementary file 1 — Supplementary Material 1: Table S1. ddPCR results of series dilution of MYD88 L265P; Table S2. ddPCR results of series dilution of CD79B Y196; Table S3. ddPCR results of healthy controls and patient samples; Figure S1. cfDNA yields of plasma samples from PCNSL patients; Figure S2. Series dilution VAF of MYD88 L265P and CD79B Y196. [file 12885_2024_12191_MOESM1_ESM.pdf]

Table S1. ddPCR results of postive control and negative control of MYD88 L265P

Sample	ddPCR for MYD88 L265P		
	Mutant droplets	Total filled droplets	VAF
**Series dilution 1**			
Expected VAF 10%	516	3756	0.1374
Expected VAF 5%	292	3777	0.0773
Expected VAF 1%	54	3316	0.0163
Expected VAF 0.5%	37	3707	0.0100
Expected VAF 0.3%	26	3527	0.0074
Expected VAF 0.1%	5	3596	0.0014
**Series dilution 2**			
Expected VAF 10%	470	3496	0.1344
Expected VAF 5%	262	3858	0.0679
Expected VAF 1%	56	3687	0.0152
Expected VAF 0.5%	29	3180	0.0091
Expected VAF 0.3%	19	3823	0.0050
Expected VAF 0.1%	6	4065	0.0015
**Series dilution 3**			
Expected VAF 10%	587	3913	0.1500
Expected VAF 5%	243	3586	0.0678
Expected VAF 1%			FAIL
Expected VAF 0.5%	34	3897	0.0087
Expected VAF 0.3%	20	3611	0.0055
Expected VAF 0.1%	9	3345	0.0027

Table S2. ddPCR results of postive control and negative control of CD79B Y196

Sample	ddPCR for CD79B Y196		
	Mutant droplets	Total filled droplets	VAF
**series dilution1**			
Expected VAF 10%	102	1120	0.0911
Expected VAF 5%	116	1888	0.0614
Expected VAF 1%	25	2456	0.0102
Expected VAF 0.5%	23	3048	0.0075
Expected VAF 0.3%	5	2458	0.0020
Expected VAF 0.1%	6	2918	0.0021
**Series dilution 2**			
Expected VAF 10%	147	1280	0.1148
Expected VAF 5%	117	1892	0.0618
Expected VAF 1%	27	2697	0.0100
Expected VAF 0.5%	23	3126	0.0074
Expected VAF 0.3%	6	2593	0.0023
Expected VAF 0.1%	4	3187	0.0013
**Series dilution 3**			
Expected VAF 10%	138	1414	0.0976
Expected VAF 5%	99	1861	0.0532
Expected VAF 1%	35	2995	0.0117
Expected VAF 0.5%	13	2426	0.0054
Expected VAF 0.3%	15	3221	0.0047
Expected VAF 0.1%	4	2644	0.0015

Table S3. ddPCR results of healthy controls and patient samples

Case	Plasma sampling	Histology	Plasma collecting Tube	cfDNA yield/ml	ddPCR for MYD88 L265P					ddPCR for CD79B Y196				
					Tissue	Plasma				Tissue	Plasma			
						Mutant droplets	Total filled droplets	VAF	Result cfDNA		Mutant droplets	Total filled droplets	VAF	Result cfDNA
#1	Diagnosis	PCNSL	EDTA	4.2	negative	1	729	0.0014	negative	negative	0	372	0.0000	inconclusive
#2	Diagnosis	PCNSL	STRECK	14	positive	1	685	0.0015	negative	positive	1	779	0.0013	negative
#3	Diagnosis	PCNSL	STRECK	15.5	negative	0	1221	0.0000	negative	negative	0	1095	0.0000	negative
#4	Diagnosis	PCNSL	STRECK	12	positive	2	2312	0.0009	negative	negative	2	931	0.0021	negative
#5	Diagnosis	PCNSL	STRECK	34.5	positive	4	2423	0.0017	negative	negative	1	1595	0.0006	negative
#6	Diagnosis	PCNSL	STRECK	14.1	positive	2	1329	0.0015	negative	negative	2	902	0.0022	negative
#7	Diagnosis	PCNSL	STRECK	14	positive	1	1404	0.0007	negative	positive	1	650	0.0015	negative
#8	Diagnosis	PCNSL	STRECK	8.9	positive	18	1774	0.0101	positive	negative	0	654	0.0000	negative
#9	Diagnosis	PCNSL	EDTA	26		3	1254	0.0024	negative		1	1267	0.0008	negative
#10	Diagnosis	PCNSL	STRECK	14.9		0	1349	0.0000	negative		0	525	0.0000	negative
#11	Diagnosis	PCNSL	STRECK	18.9		0	2780	0.0000	negative		1	1284	0.0008	negative
#12	Diagnosis	PCNSL	STRECK	10.8		1	1494	0.0007	negative		0	740	0.0000	negative
#13	Diagnosis	PCNSL	STRECK	10.8		0	1266	0.0000	negative		0	628	0.0000	negative
#14	Diagnosis	PTLD-PCNSL	EDTA	19.9		0	1232	0.0000	negative		0	963	0.0000	negative
#15	PD	PCNSL	EDTA	65		7	1949	0.0036	positive		2	1266	0.0016	negative
#16	PD	PCNSL	STRECK	8	negative	0	462	0.0000	inconclusive		0	620	0.0000	negative
#17	PR	PCNSL	EDTA	6.6	positive	0	1134	0.0000	negative	negative	0	258	0.0000	inconclusive
#18	PR	PCNSL	EDTA	7	positive	7	1278	0.0055	positive	negative				not available
#19	PR	PCNSL	EDTA	10.1		0	1095	0.0000	negative		0	733	0.0000	negative
#20	PR	PCNSL	STRECK	10.9		2	2085	0.0010	negative		1	886	0.0011	negative
#21	PR	PCNSL	STRECK	16	positive	4	2470	0.0016	negative	negative	0	384	0.0000	inconclusive
#22	PR	PCNSL	STRECK	8	negative	0	878	0.0000	negative	negative	0	406	0.0000	inconclusive
#23	PR	PTLD-PCNSL	STRECK	44.5		0	1831	0.0000	negative		0	1085	0.0000	negative
#24	PR	PTLD-PCNSL	STRECK	16.1	negative	0	1479	0.0000	negative	negative	0	815	0.0000	negative
**WBC**														
1	WBC					0	3313	0.0000	negative		4	3073	0.0013	negative
2	WBC					0	1569	0.0000	negative		3	1587	0.0019	negative
3	WBC					0	917	0.0000	negative		0	836	0.0000	negative
4	WBC					1	4218	0.0002	negative		0	4058	0.0000	negative
5	WBC					0	1378	0.0000	negative		0	1276	0.0000	negative
6	WBC					0	710	0.0000	negative		1	816	0.0012	negative
7	WBC					2	5765	0.0003	negative		1	5318	0.0002	negative
8	WBC					1	1292	0.0008	negative		3	2493	0.0012	negative
9	WBC					1	2723	0.0004	negative		0	1024	0.0000	negative
10	WBC					2	4465	0.0004	negative		2	3717	0.0005	negative
11	WBC					2	1383	0.0014	negative		0	1292	0.0000	negative
12	WBC					0	661	0.0000	negative		2	584	0.0034	negative
13	WBC					0	2381	0.0000	negative		2	2085	0.0010	negative
14	WBC					1	500	0.0020	negative		0	511	0.0000	negative
15	WBC					0	324	0.0000	inconclusive		1	275	0.0036	inconclusive
16	WBC					1	4622	0.0002	negative		1	4422	0.0002	negative
17	WBC					0	1581	0.0000	negative		0	1427	0.0000	negative
18	WBC					1	847	0.0012	negative		0	797	0.0000	negative
19	WBC					1	2891	0.0003	negative		3	3211	0.0009	negative
20	WBC					1	1592	0.0006	negative		0	1578	0.0000	negative
21	WBC					0	768	0.0000	negative		1	871	0.0011	negative
22	WBC					1	3907	0.0003	negative		1	3179	0.0003	negative
23	WBC					1	2114	0.0005	negative		0	2021	0.0000	negative
24	WBC					1	987	0.0010	negative		0	960	0.0000	negative
**Controls**														
1	Positive control_1					3050	3051	0.9997	positive		348	1129	0.3082	positive
2	Positive control_2					1682	1682	1.0000	positive		231	706	0.3272	positive
3	Negative control_1					0	3566	0.0000	negative		3	3253	0.0009	negative
4	Negative control_2					0	2087	0.0000	negative		1	1654	0.0006	negative
5	No template control_1					0	0				0	0		
6	No template control_2					0	0				0	0		

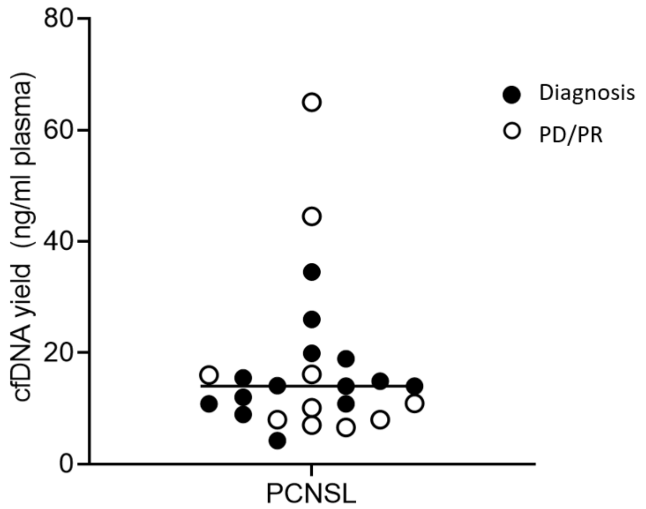

Figure S1. cfDNA yields of plasma samples from PCNSL patients. DNA yields ranged from 4.4 to 65 ng per ml of plasma. The median DNA yield in PCNSL cases was 14 ng/ml (range 4.4 to 65 ng/ml). Partial response (PR); Progressive disease (PD).

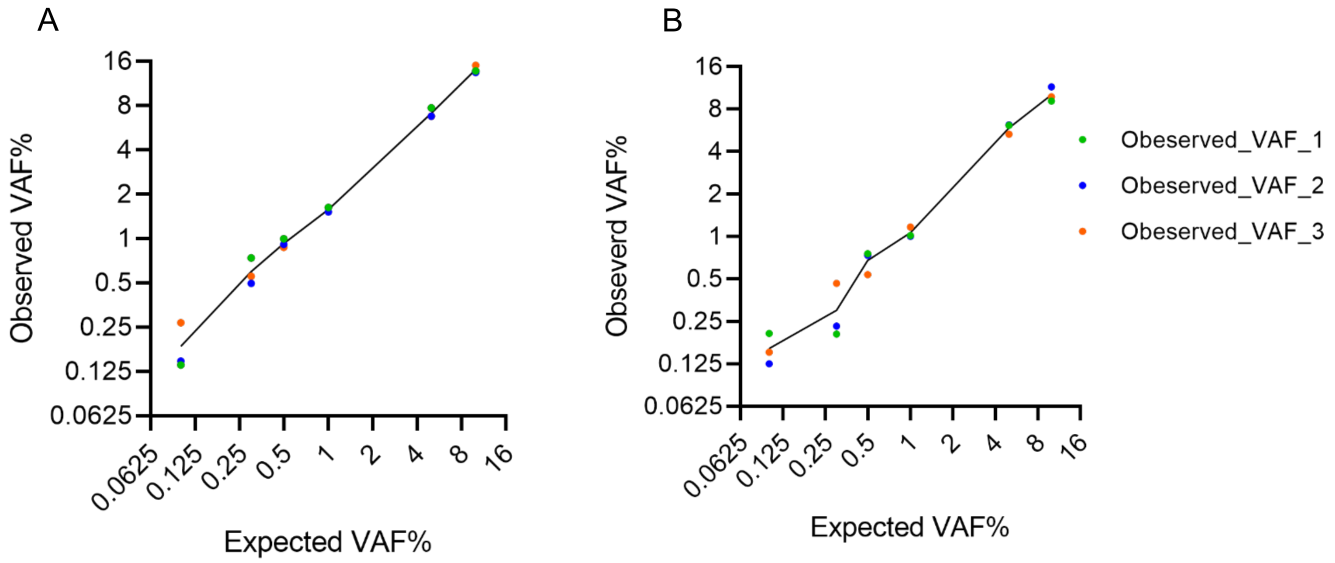

Figure S2. Stimulate VAF of MYD88 L265P and CD79B Y196. A. ddPCR test of MYD88 L265P on three independent series dilutions, expected VAFs is 10%, 5%, 1%, 0.5%, 0.3%, 0.1%. Observed results show a clear linear pattern ranging from 14% to 0.25%. B. ddPCR test of CD79B Y196 on three independent series dilutions, expected VAFs is 10%, 5%, 1%, 0.5%, 0.3%, 0.1%. Observed results show a clear linear pattern ranging from 10% to 0.5%. The black line shows the average VAF of the three independent experiments.
